# Prediction of the development of islet autoantibodies through integration of environmental, genetic, and metabolic markers

**DOI:** 10.1111/1753-0407.13093

**Published:** 2020-08-16

**Authors:** Bobbie‐Jo M. Webb‐Robertson, Lisa M. Bramer, Bryan A. Stanfill, Sarah M. Reehl, Ernesto S. Nakayasu, Thomas O. Metz, Brigitte I. Frohnert, Jill M. Norris, Randi K. Johnson, Stephen S. Rich, Marian J. Rewers

**Affiliations:** ^1^ Biological Sciences Division, Pacific Northwest National Laboratory Richland Washington USA; ^2^ Colorado School of Public Health University of Colorado Anschutz Medical Campus Aurora California USA; ^3^ Computing and Analytics Division Pacific Northwest National Laboratory Richland Washington USA; ^4^ Barbara Davis Center for Diabetes University of Colorado Anschutz Medical Campus Aurora Colorado USA; ^5^ Center for Public Health Genomics University of Virginia Charlottesville Virginia USA

**Keywords:** autoimmunity, genetics, machine learning, metabolomics, 自身免疫, 遗传, 机器学习, 代谢组学

## Abstract

**Background:**

The Environmental Determinants of the Diabetes in the Young (TEDDY) study has prospectively followed, from birth, children at increased genetic risk of type 1 diabetes. TEDDY has collected heterogenous data longitudinally to gain insights into the environmental and biological mechanisms driving the progression to persistent islet autoantibodies.

**Methods:**

We developed a machine learning model to predict imminent transition to the development of persistent islet autoantibodies based on time‐varying metabolomics data integrated with time‐invariant risk factors (eg, gestational age). The machine learning was initiated with 221 potential features (85 genetic, 5 environmental, 131 metabolomic) and an ensemble‐based feature evaluation was utilized to identify a small set of predictive features that can be interrogated to better understand the pathogenesis leading up to persistent islet autoimmunity.

**Results:**

The final integrative machine learning model included 42 disparate features, returning a cross‐validated receiver operating characteristic area under the curve (AUC) of 0.74 and an AUC of ~0.65 on an independent validation dataset. The model identified a principal set of 20 time‐invariant markers, including 18 genetic markers (16 single nucleotide polymorphisms [SNPs] and two HLA‐DR genotypes) and two demographic markers (gestational age and exposure to a prebiotic formula). Integration with the metabolome identified 22 supplemental metabolites and lipids, including adipic acid and ceramide d42:0, that predicted development of islet autoantibodies.

**Conclusions:**

The majority (86%) of metabolites that predicted development of islet autoantibodies belonged to three pathways: lipid oxidation, phospholipase A2 signaling, and pentose phosphate, suggesting that these metabolic processes may play a role in triggering islet autoimmunity.

## Graphical abstract

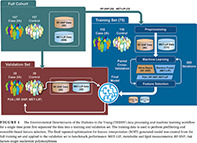

**Highlights**

- A machine learning model can significantly predict imminent development of islet autoimmunity based on environmental, genetic, and metabolic features.
- The machine learning algorithm feature selection identified type 1 diabetes‐associated single nucleotide polymorphisms from The Environmental Determinants of the Diabetes in the Young (TEDDY) analysis are correlated to related data from the Diabetes Autoimmunity Study in the Young (DAISY) study.
- Most of the metabolic features predicting the development of islet autoantibodies belonged to three pathways; lipid oxidation, phospholipase A2 signaling, and pentose phosphate.

## 摘要

**背景:**

青少年糖尿病的环境决定因素(TEDDY)研究一直跟踪着1型糖尿病遗传风险增加的儿童从出生到目前的状况。 TEDDY纵向收集了各种数据，以深入了解推动胰岛自身抗体发展的环境和生物学机制。

**方法:**

我们基于时变代谢组学数据并结合时变危险因素(例如胎龄)，开发了一种机器学习模型来预测持续性胰岛自身抗体的发展。机器学习最初具有221种潜在特征(包括85种遗传特征，5种环境特征，131种代谢组学特征)，并且基于整体的特征评估用于识别一小部分预测特征，可以对其进行查询以更好地了解导致持续性胰岛自身免疫的发病机理。

**结果:**

最终的集成机器学习模型包括42个不同特征，交叉验证的受试者工作特征曲线下的面积(AUC)为0.74，一个独立验证数据集的AUC约为0.65。该模型确定了一组主要的20个非时变标记，包括18个遗传标记(16个单核苷酸多态性[SNPs]和两个HLA‐DR基因型)和2个人口统计学标记(胎龄和使用益生元)。通过与代谢组整合鉴定出22种补充代谢产物和脂质，包括己二酸和神经酰胺d42:0，这些物质可预测胰岛自身抗体的产生。

**结论:**

预测胰岛自身抗体产生的大多数(86%)代谢产物属于三个途径:脂质氧化途径、磷脂酶A2信号传导和磷酸戊糖途径，表明这些代谢过程可能在触发胰岛自身免疫中起作用。

## 1 INTRODUCTION

The risk of type 1 diabetes (T1D) involves both genetic and nongenetic factors. Our understanding of the role of human leukocyte antigen (HLA) and other genes in development of islet autoimmunity and subsequent progression to T1D is continually expanding.^1^, ^2^, ^3^ Recent work has focused on understanding how these genetic factors interact with environmental factors and biomarkers of T1D risk.^4^, ^5^, ^6^, ^7^ Better prediction of the risk of T1D incorporating multiple predictive factors to offer new strategies for early diagnosis and treatment is one of the core goals of birth cohort studies studying genetically susceptible children, such as The Environmental Determinants of Diabetes in the Young (TEDDY) and Diabetes Auto Immunity Study in the Young (DAISY). Herein we focus on prediction of imminent progression to the development of persistent islet autoantibodies to insulin (IAA), glutamic acid decarboxylase (GADA), or insulinoma antigen‐2 (IA‐2A) in TEDDY participants by integrating data such as metabolomics that are measured within 6 months prior to the diagnosis of persistent autoimmunity with associated risk factors, such as genetics and environment.

Identification of robust molecular markers from large and complex data has been noted as one of the major challenges of personalized medicine.^8^, ^9^ One strategy is to utilize a knowledge‐based approach, where known risk factors for a disease are combined in a machine learning framework to make individualized predictions. Alternatively, a data‐driven approach can be taken that allows potential markers, such as metabolite characterization from high‐throughput 'omic studies, to be incorporated into the model and further interrogated to identify an optimal subset of risk factors to make statistical‐based predictions of interest. In recent studies we took the latter approach to evaluate a small cohort from DAISY at various time points prior to the development of persistent autoantibodies Islet autoimmunity (IA) to both predict phases of development and also to identify the core features of importance.^10^ In this study, we expand on this approach to perform machine learning‐based ensemble feature selection on 314 children who had metabolomic data across time in the TEDDY nested case‐control study.^11^ We utilize a probability‐based machine learning integration strategy combined with an optimization‐based feature selection process to identify a core set of predictive markers. We further examine the core features that drive the machine learning model and assess the mechanistic changes associated with these features. We present results in the context of cross‐validation and an independent holdout set.

## 2 METHODS

### 2.1 Participants selection and data generation

The TEDDY study includes 8676 participants with increased T1D risk HLA‐DR/DQ genotypes, recruited before the age of 4.5 months from four countries; United States, Germany, Sweden, and Finland.^12^ The children were evaluated for the development of persistent islet autoantibodies every 3 months until either the development of T1D or 48 months. At this point for those with autoantibody seroconversion visits continue every 3 months, and for the rest, visits proceed at 6‐month intervals. All study participants had written informed consent from a parent or primary caretaker. The TEDDY study was approved by the local institutional review boards where the data were collected and is monitored by an external evaluation committee formed by the National Institutes of Health (NIH).

### 2.2 Islet autoantibody measurements

IAA, GADA, or IA‐2A were measured in two laboratories by radiobinding assays as previously described.^13^, ^14^ In the United States, all sera were assayed at the Barbara Davis Center for Diabetes at the University of Colorado Denver; in Europe, all sera were assayed at the University of Bristol, United Kingdom. Both laboratories have previously shown high sensitivity and specificity^14^ as well as concordance. To optimize concordance, harmonized assays for GADA and IA‐2A (12) replaced previous assays, in January 2010. Based on a receiver‐operator curve (ROC) analysis, prior samples that needed to be reanalyzed with the harmonized assays, included Denver GADA between −0.015 and 0.042; Bristol GADA between 10.69 and 36.72; Denver IA‐2A between −0.004 and 0.016; and Bristol IA‐2A between 6.69 and 10.58. All positive islet autoantibodies and 5% of negative samples were retested in the other reference laboratory and deemed confirmed if concordant.

### 2.3 Outcomes

Persistent confirmed islet autoimmunity (IA) was defined as two consecutive visits positive for a specific islet autoantibody confirmed in a second laboratory. Date of IA was the draw date of the first sample of the two consecutive samples that deemed the child persistent confirmed positive for any islet autoantibody.

### 2.4 Nested case‐control study

Because of the large cohort size, it is cost and time prohibitive to perform many 'omics‐based analyses, such as microbiome^15^, ^16^ and metabolomics,^17^ for all the samples. The case‐control study design can also help to reduce the batch effects associated with large‐scale 'omics studies. The TEDDY Data Coordinating Center generated nested case‐control pairs using a design that is based on the time point at which a child is positive for an event. The event is either the presentation of persistent islet autoantibodies or clinical diagnosis of T1D. Once a case is defined, an associated control is selected from all event‐free participants at that same time point for the case. The best control for the case is selected based on matching factors of clinical center, sex, and family history of T1D.^11^, ^18^, ^19^ In the analyses presented here, we included 157 cases who developed persistent islet autoantibodies and 157 matched controls, a 1:1 nested case‐control design described in detail by Lee et al.^11^

### 2.5 Data sources

The predictive model developed here was based on three sources of data: (a) participant risk factors (RF) previously identified in the study population,^6^, ^11^ (b) participant genetic risk (T1D‐associated single nucleotide polymorphisms [SNPs]^3^), and (c) participant metabolomic risk (metabolites and lipids). The goal of this modeling effort is the prediction of progression to persistent autoimmunity, thus all data used is either independent to or collected prior to the observance of autoantibodies.

The first data source was a combination of participant risk factors, including genetic features, associated with T1D risk (RF‐SNP). The patient risk factors were selected based on their availability in the case‐control data and included (a) gestational age in weeks, (b) exposure to cow's milk formula, (c) exposure to prebiotic formula, (d) ethnicity/race (white, unknown, multiracial), and (e) HLA risk genotypes (DR3/4, DR4/4, DR4/8, DR4/1, DR4/13, DR3/3) previously described in.^5^ Except for gestational age, each of these were represented as binary variables and thus in the dataset is represented as 12 variables. The T1D‐associated SNP data were generated using a custom genotyping array (Illumina ImmunoChip) containing 186 000 SNPs associated with autoimmune disease. The data were collected for all TEDDY individuals with genotyping conducted by the TEDDY Genetics Laboratory at the University of Virginia Center for Public Health Genomics. A total of 85 SNPs significantly associated with T1D were used in this analysis.^3^, ^20^

The second source of data was time‐varying metabolite and lipid measurements (MET‐LIP) from participants at time points prior to the development of persistent autoimmunity. Untargeted metabolomics and lipidomics data were generated for all cases and controls in the TEDDY nested case‐control study. Primary metabolites and lipids were quantified from citrated plasma using gas chromatography‐time‐of‐flight mass spectrometry (GC‐TOF MS) and charged surface hybrid liquid chromatography coupled to quadrupole TOF MS (CSH‐QTOF MS),^21^ respectively, at the NIH West Coast Metabolomics Center at the University of California, Davis, California. The GC‐TOF MS metabolomics data acquisition followed previously described protocols^22^ followed by data processing and compound identification using the BinBase algorithm^23^ and normalization using the sum approach. There were 156 identified metabolites quantified for analysis. Metabolites with less than 10% missing values were processed with random forest imputation^24^ and those with 10% or more missing data were removed. For complex lipids, samples were extracted by methyl‐tert‐butyl ether/methanol/water and analyzed using CSH‐QTOF MS in both positive and negative electrospray ionization (ESI). Lipid chromatogram peak detection and alignment used Mass Profiler Professional (Agilent, Santa Clara, CA). Peaks detected in at least 30% of samples were identified and quantification back‐filled using the Fiehn laboratory's LipidBlast spectral library.^25^ Locally weighted scatter plot smoother (LOESS)‐based normalization was corrected for batch effects by adjusting individual samples to intermittent quality control samples.^26^ There were 652 lipids identified across both positive and negative ESI modes.

### 2.6 Integrative machine learning

We built machine learning models to predict cases vs controls at three time horizons to the development autoantibodies: (a) 0 months (the time at which positive autoantibodies were first detected for cases), (b) 3 months prior, and (c) 6 months prior. Data were assembled by identifying the age of the case at the time of being classified as autoantibody positive and selecting the sampling time point for the control that matched the age of the case as close as possible. In addition, there are situations in which a child is identified as both a control and a case because of the risk set sampling used for the nested case control study design from the longitudinal study. These situations were removed from the dataset because they cause issues with the independence assumptions between subjects of the machine learning models. Most children are sampled approximately every 3 months and, thus, we used data that represent the case and control pair at the two prior sampling time points based on the age of the case at confirmation of autoimmunity. The point of seroconversion (0 months) is included to evaluate if the prediction of imminent progression can be achieved with similar accuracy once autoantibodies are detectable.

Of all case‐control pairs available for the autoimmunity endpoint, there were 314 children (157 pairs) that contained the RF‐SNP and MET‐LIP data at all three time points. We segregated a validation set prior to machine learning consisting of 25% of the pairs (78 total children) with the remaining 236 children utilized as the training set. Descriptive statistics of the training and validation sets are shown in Supplemental Table S1. The 39 pairs in the validation set were selected at random but have similar overall characteristics as the training set (last column of Table S1).

**FIGURE 1:**
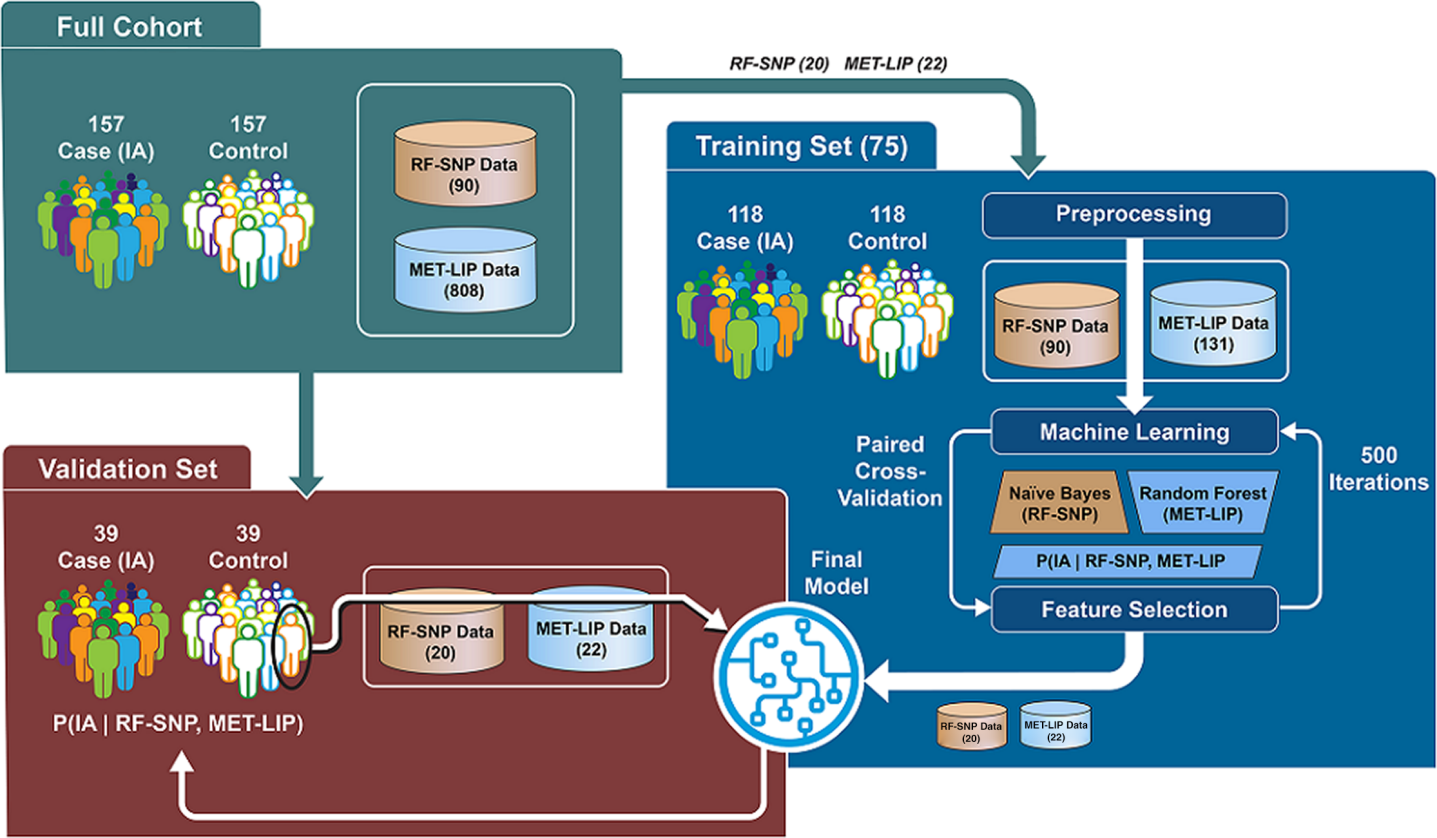
The Environmental Determinants of the Diabetes in the Young (TEDDY) data processing and machine learning workflow for a single time point first separated the data into a training and validation set. The training data is used to perform prefiltering and ensemble‐based feature selection. The final repeated optimization for feature interpretation (ROFI) generated model was created from the full training set and applied to the validation set to benchmark performance. MET‐LIP, metabolite and lipid measurements; RF‐SNP, risk factors‐single nucleotide polymorphisms

The workflow of the analysis performed is shown in Figure 1. The first step separated the validation data from the training data to ensure that data quality and filtering is independent of the validation set and will not bias the downstream machine learning evaluation. Once the validation set was segregated, the data were preprocessed and the model was developed. As is common for machine learning,^27^ the metabolites and lipids were preprocessed with a paired *t* test (comparing cases to controls) in the training set at each time point to reduce the dimensionality before machine learning. A conservative minimum significance threshold of *P* = 0.1 across the time points was selected; this yielded 131 markers consisting of 45 metabolites and 86 lipids. The metabolites and lipids in the validation set were reduced to match the training set but were not utilized in generating the statistics for the down‐selection criteria.

Multiple machine learning algorithms, including logistic regression, K‐nearest neighbors, linear discriminant analysis, Naïve Bayes classifier, random forest, and a support vector machine (linear kernel), were completed on the base level RF‐SNP features and MET‐LIP features of with five‐fold cross‐validation (CV) to evaluate which machine learning algorithm best modeled the underlying structure of the data. A random forest approach was selected for the MET‐LIB data (131 metabolome features) and a Naïve Bayes classifier for the RF‐SNP (90 SNP/environmental features). The models were merged as the product of the posterior probability from each machine learning algorithm to attain a single probabilistic score for each child.^28^, ^29^ We validated that the merged model was equal to or superior to combining the two sources of data into a single Naïve Bayes or random forest model. At seroconversion the merged model returned significantly larger ROC area under the curve (AUC)s than either a single Naïve Bayes or random forest model based on 100 repetitions of fivefold CV at a paired *t* test *P*‐value threshold of 0.05.

Repeated optimization for feature interpretation (ROFI) generated the features that optimize the ability to separate those that will transition to IA positivity in the three defined time windows.^10^, ^30^ ROFI performs an optimization‐based feature selection algorithm repeated 500 times and the importance of a feature is defined as the percentage of times it was selected for inclusion in the model over the 500 independent optimization runs based on five‐fold CV. Given the paired nature of the nested case‐control study, the CV process ensured that pairs were placed together in a training or a holdout set in the CV in order to reduce bias from potential pairwise correlation. Once the feature importance metrics were acquired, the values were sorted and the features to be included in the final model development were selected. The final model was generated on the full training data of 236 TEDDY children based on the features selected from ROFI; the machine learning model was applied to the validation set to generate the likelihood that each of the 78 children to develop persistent autoimmunity with the defined time frame, with the full process repeated for each of the three time points (Figure 1). The machine learning algorithms and feature selection method are available at https://github.com/pmartR/peppuR.

## 3 RESULTS

The data here were derived from the nested case‐control design of TEDDY and, therefore, features of family history, sex, clinical center, and age are not included in the model because they are utilized as matching criteria. The machine learning model generates the probability that a TEDDY child will transition from a control state to the development of persistent autoantibodies within the next *x* months (*x* = 0, 3, 6) based on the defined risk factors, genetic profile, and metabolome. The average age of the children that developed autoantibodies, “cases,” was ~2 years old (range from 0.72 to 4 years); thus, predictions for children not in this age range may not be applicable.

### 3.1 Feature selection and performance

**FIGURE 2:**
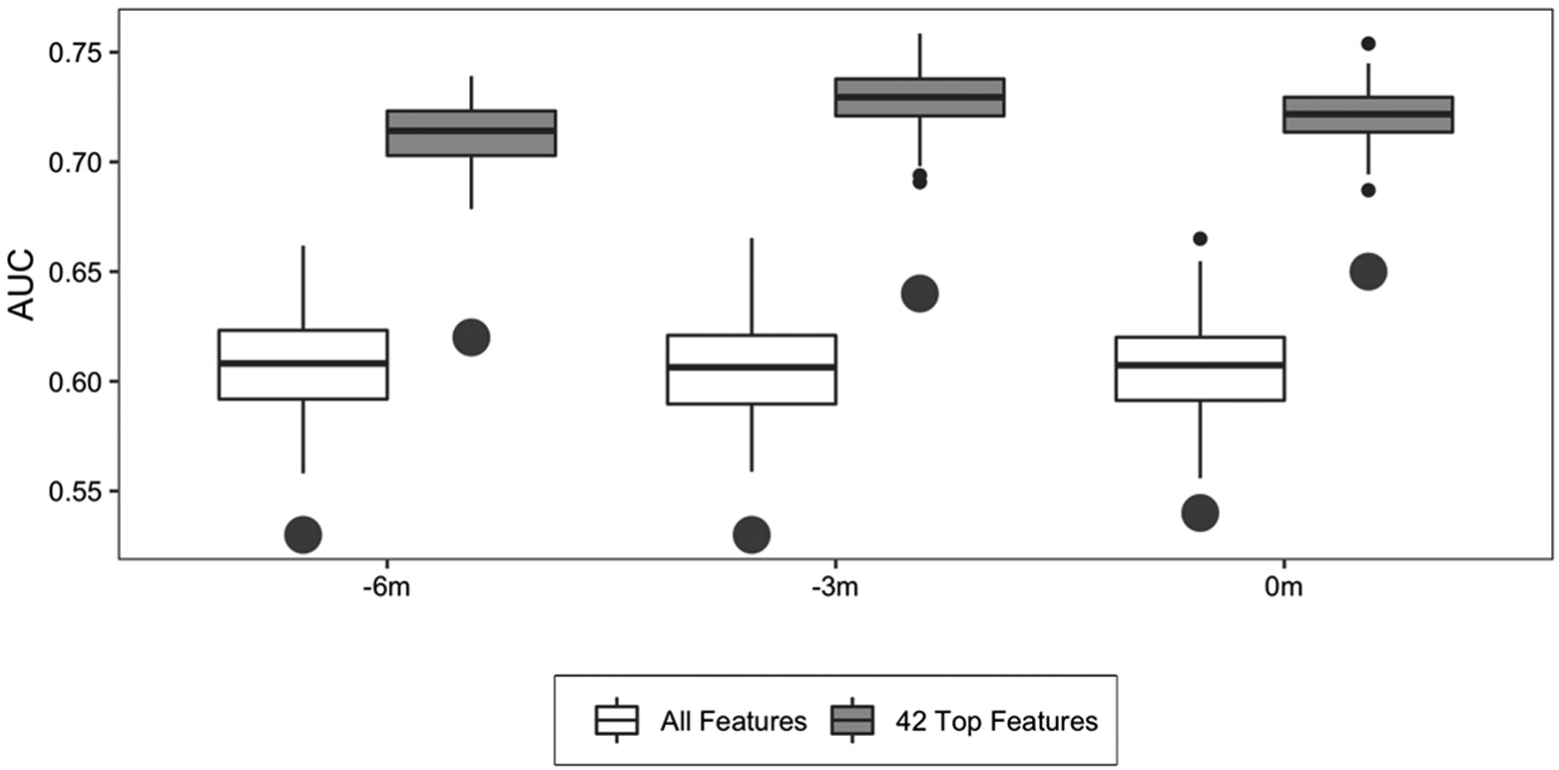
Overall accuracy and variability of the training data (boxplots) based on cross‐validation and the associated accuracy of the model when applied to the validation set (large dots) for both all features and the 42 selected features. The boxes of the training data results represent the 25th and 75th percentiles and the line indicates the median accuracy with extreme values represented by the small dots. AUC, area under the curve

**FIGURE 3:**
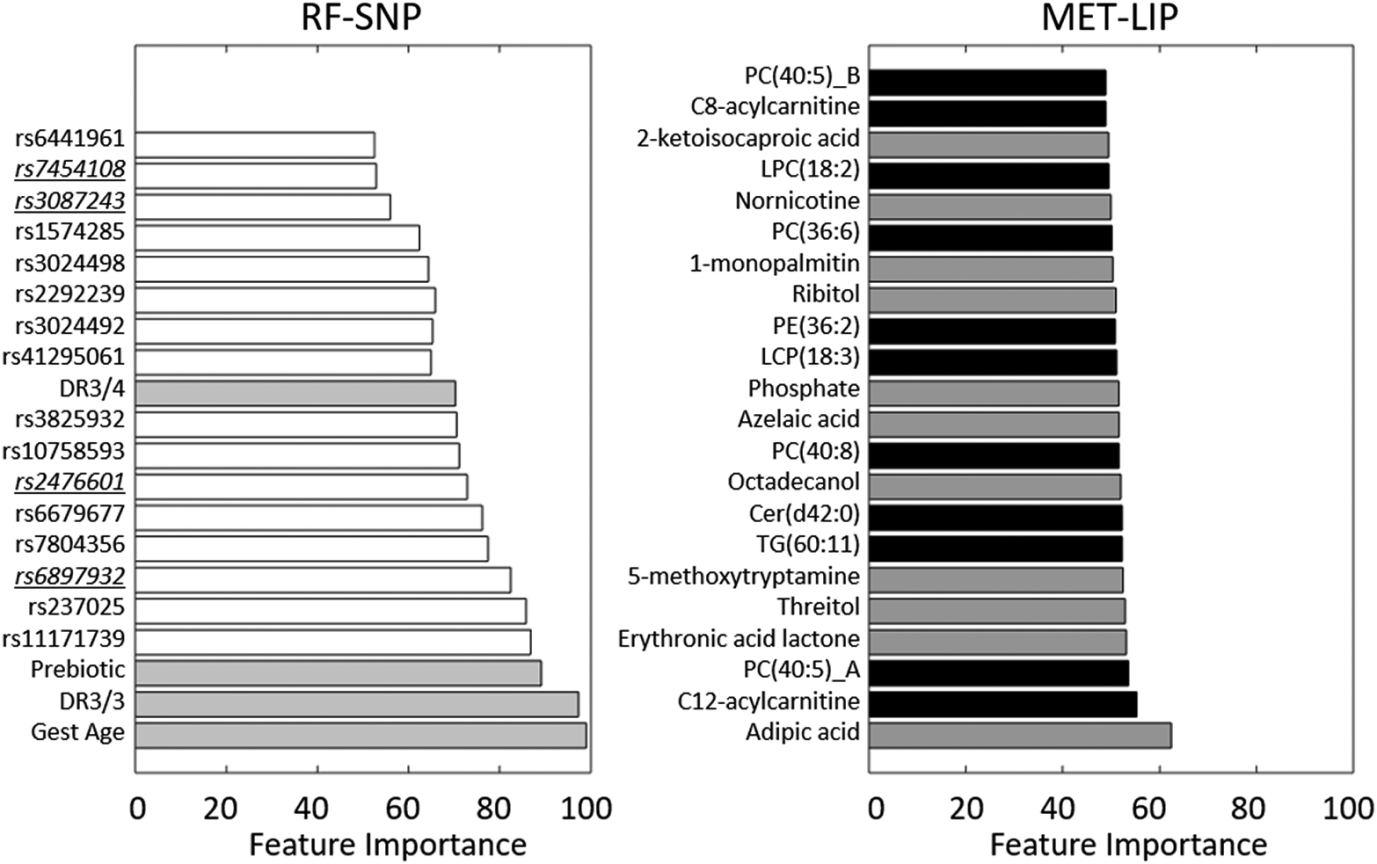
Bar graphs showing the feature importance of each of the selected 42 candidates where time‐invariant markers are on the left with single nucleotide polymorphisms (SNPs) in white and other risk factors in gray and metabolomics data on the right where lipids are in black and metabolites in gray. The underlined SNPs are those highlighted in Figure 5. MET‐LIP, metabolite and lipid measurements; RF‐SNP, risk factors‐single nucleotide polymorphisms

We performed ROFI‐based feature selection on 131 metabolome features, 85 T1D‐associated SNPs features, in addition to five features representing HLA category, gestational age, cow's milk formula exposure, prebiotic formula exposure, and ethnicity/race. The RF‐SNP data was the same at each time point, but the metabolome is represented as three datasets of quantitative metabolite and lipid values at 0, −3, and ‐6 m from seroconversion. The feature selection process identified 42 features as optimal utilizing an order‐based statistic to define the selection threshold.^31^ As seen in Figure 2 there is a dramatic improvement for both the cross‐validated training data (boxplots) and the associated accuracy of the validation set (point estimate) at all time points for the feature selection model vs using all 221 features. Of these 42 features 20 were associated with the risk factors and genetic markers, and the remaining 22 were from the metabolome (11 metabolites and 11 lipids). Figure 3 displays the importance, defined as the percentage of solutions including the feature, of each these 42 candidates identified by ROFI. Figure 2 also demonstrates that the prediction of imminent development can roughly be predicted with the same accuracy 3 and 6 months prior to the time point when the autoantibodies are observed.

### 3.2 Development of IA: Machine learning

**FIGURE 4:**
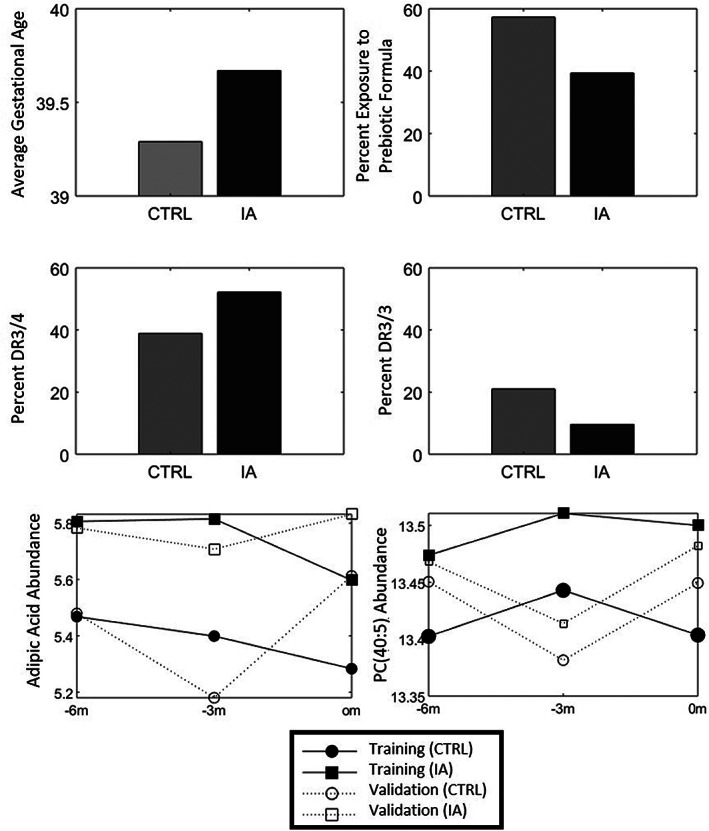
Data graphs showing the directional changes of (A) gestational age, (B) exposure to prebiotic formula, (C) DR3/4 and (D) DR3/3, as well as the temporal changes for (E) adipic acid and (F) phosphotidycholine PC(40:5) for the IA and associated control samples (CTRL)

Gestational age is one of potential risk factors for T1D reported previously.^32^ For the training set, the average gestational age of cases was 0.48 weeks longer than that of the controls (*P ~* 0.008) that decreases to a statistically insignificant difference of 0.09 weeks in the validation set (*P ~* 0.811), Figure 4A. It is not clear whether the result in the validation set is due to low statistical power or random fluctuation in the training/validation process. Figures 4B‐D show the other three important risk factors ‐ prebiotic exposure, HLA‐DR3/4, and HLA‐DR3/3 genotypes ‐follow expected patterns^4^, ^33^ and are more strongly correlated between the training and validation sets. The metabolite with the strong feature importance score (adipic acid) and one of the top lipids (PC[40:5]) separate patterns across time for the full cohort, training, and validation sets for the T1D case‐control matched pairs (Figure 4E, Figure 4F). These markers have been associated with diabetes^34^, ^35^, ^36^ and patterns of increased abundance of these metabolites appear to predict the development of persistent autoantibodies in the TEDDY population.

**FIGURE 5:**
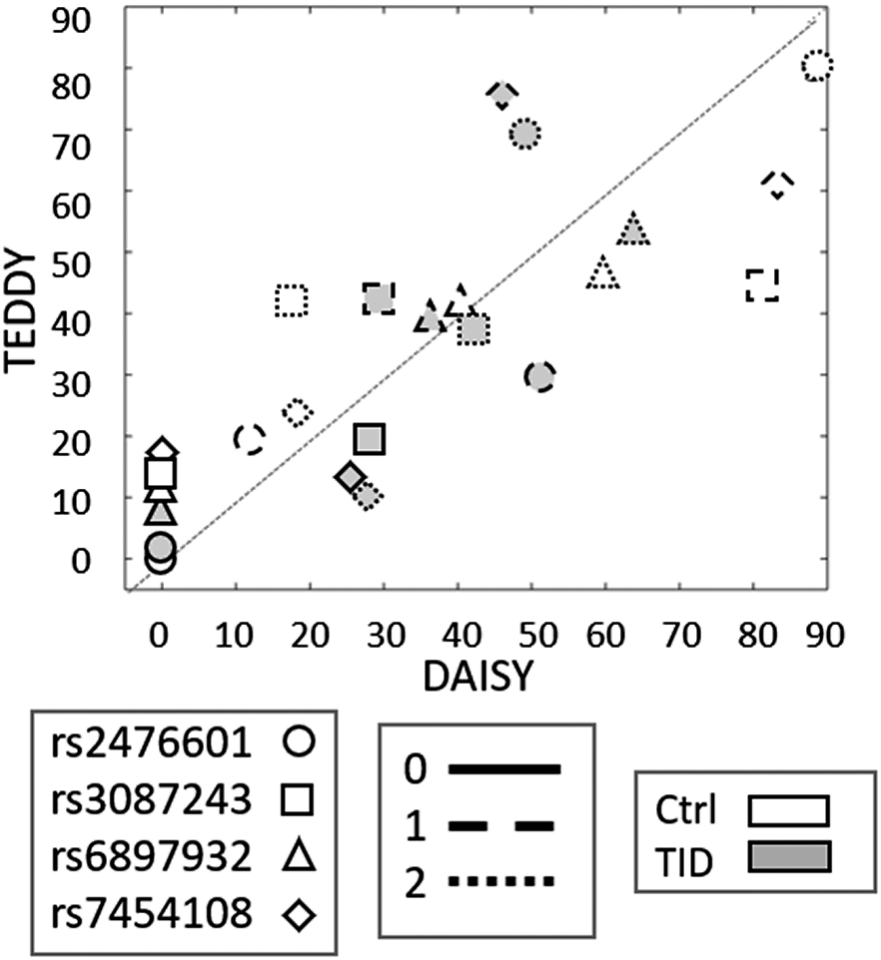
Scatter plot of percentage of children in each genotype for four of the top single nucleotide polymorphisms (SNPs) in terms of feature importance that overlap with SNPs currently being studied in the DAISY cohort. DAISY, Diabetes Auto Immunity Study in the Young; TEDDY, The Environmental Determinants of the Diabetes in the Young; T1D, type 1 diabetes

There were 16 SNPs that were identified by the machine learning approach that were able to distinguish the cases from controls for the development of autoantibodies. Because DAISY is a distinct cohort with similar goals to TEDDY as well as having genetic data on a subset of participants, we evaluated wheather the SNPs identified in this analysis from TEDDY have a similar pattern in DAISY. In particular, DAISY is a longitudinal, observational birth cohort study of 2547 high‐risk children followed to development of autoimmunity and T1D.^37^, ^38^, ^39^ For DAISY, genetic information was collected on 25 children that either ended the study disease free or with confirmed persistent autoantibodies. There were four SNPs that overlapped the two studies (underlined in Figure 3), for which Figure 5 shows that the minor allele frequency (MAF) of the SNPs is similar between TEDDY and DAISY (eg, rs246601 MAF in DAISY is 0.12 and MAF in TEDDY is 0.19). We estimated the correlation within each group (“case” or control) within each SNP and demonstrated that the control samples had an average Pearson correlation of 0.921 and the case samples of 0.802 (average 0.862), a confirmation that these core T1D‐associated SNPs appear to be robustly associated with outcome across studies.

**FIGURE 6:**
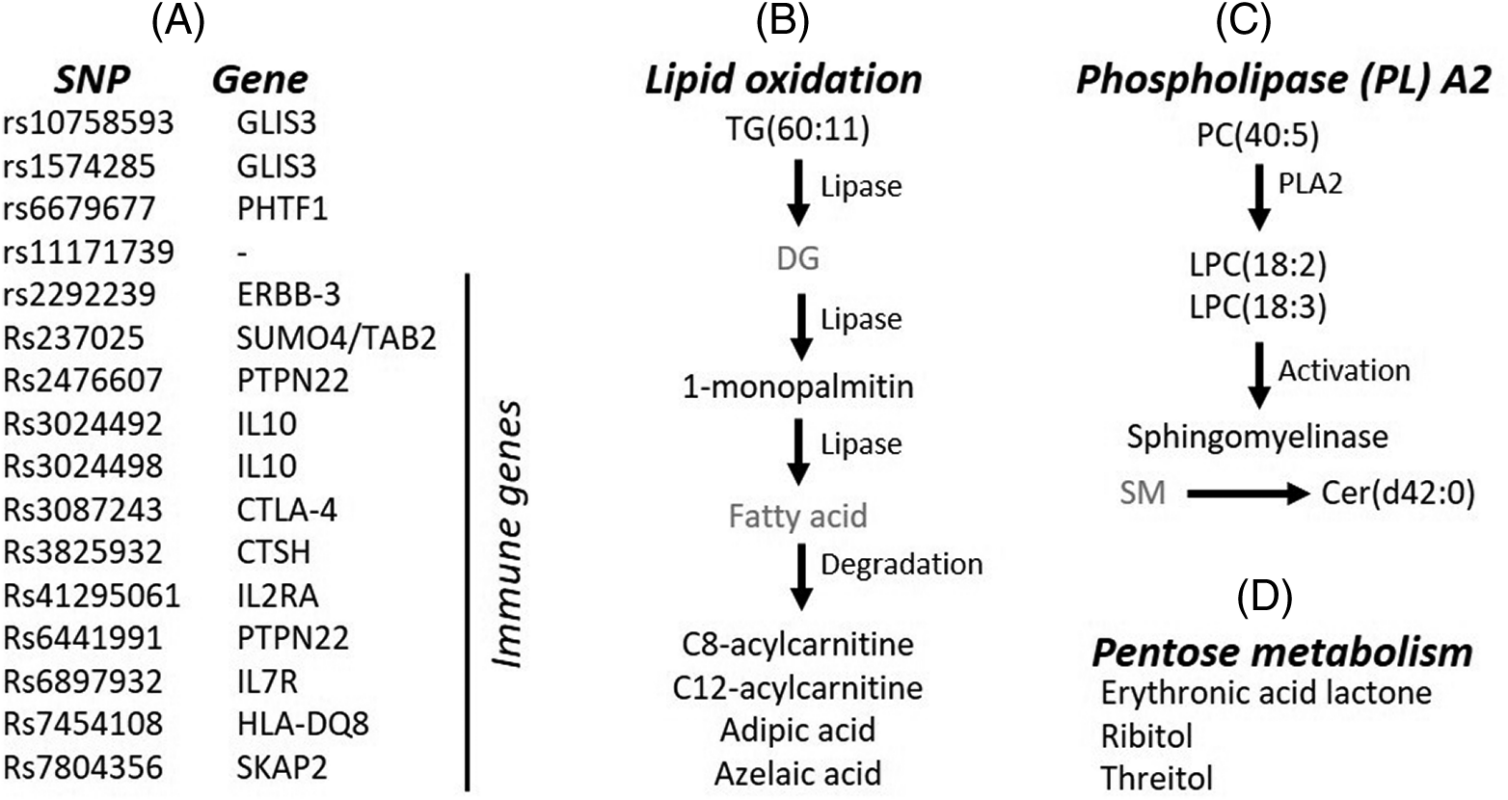
Overview of the selected features and functions. (A) List of single nucleotide polymorphisms (SNPs) and their respective genes. (B‐D) Features belonging to the lipid oxidation pathway (B), phospholipase A2 (C) and pentose metabolism (D). The metabolites in gray were not selected as features but were added to the figures merely to complete the pathways

To investigate the mechanistic indicators of the feature selection, we evaluated the SNP‐defined putative T1D target genes and metabolic pathways.^40^ Twelve out of the 16 SNPs (75%) had putative target genes related to the immune system (Figure 6A). Metabolites were mainly grouped in three pathways: lipid oxidation, phospholipase signaling, and pentose phosphate. Triacylglycerol TG(60:11), 1‐monopalmitin, C8‐acylcarnitine, C12‐acylcarnitine, adipic acid, and azelaic acid are metabolites of the lipid storage and oxidation pathway (Figure 6B). The degradation of triacylglycerols have been shown to occur in inflammation, fueling the high energy demands required for this process.^41^ The second pathway with many metabolic features predictive of autoimmunity was the phospholipase A2 signaling pathway, including phosphotidycholine PC(40:5), lysophosphatidycholines LPC(18:2) and LPC(18:3), and ceramide cer(d42:0) (Figure 6C). It has been reported that a phospholipase A2 from human islets is activated by cytokines and endoplasmic reticulum (ER) stress, leading to beta‐cell death.^35^ Phospholipase activation leads to the degradation of phosphatidylcholines (PC) into lysophosphatidylcholines (LPC) and free fatty acids, which in turn activates a neutral sphingomyelinase that cleaves sphingomyelins (SM) into phosphocholines and ceramides (Cer(d42:0)) (Figure 6C). The accumulation of ceramides triggers the apoptotic cascade resulting in the death of beta cells.^34^, ^35^ The last pathway containing several metabolic features predictive of autoimmunity is the pentose metabolism (Figure 6D). Recently, the pentose phosphate pathway was shown to be regulated in peripheral blood mononuclear cells in children IA.^42^ The pentose phosphate pathway is usually repressed in immune cells to prevent damage by toxic reactive oxygen species, but it is upregulated during autoimmune responses to supply the high metabolic demands of active leukocytes.^43^ Overall, the identified metabolic features reflect processes that are regulated during an autoimmune response.

## 4 DISCUSSION

To date, the most common approaches to predicting outcomes of diabetes have focused on regression or small variable subsets selected via expert knowledge. For example, one recent study determined the likelihood that a child will progress to T1D by the age of 6 years if they have presented with persistent autoantibodies at the age of 3 years.^27^ The logistic regression model to make this prediction utilized five predictors that were selected based on known associations, such as IA‐2 antibody positivity status and hemoglobin A1c (HbA1c) level, achieving a sensitivity of 0.91 at a specificity of 0.59, yielding a ROC curve with an AUC of 0.80. The conclusion of this work was that continued developments of such models are necessary to better understand the complexity of the disease and address long‐term goals of precision diabetes.^44^, ^45^ Biomarkers hold vast potential for clinical utility both in terms of diagnosis and prognosis of disease but also in drug discovery.^46^, ^47^

An alternate strategy to regression modeling is to take a data‐driven approach to allow a large collections of potential risk factors and molecular markers to be integrated into the predictive modeling. These machine learning models are amenable to the extraction of features that can provide improved prediction.^30^, ^48^, ^49^ Frohnert et al used a machine learning approach to mine large multi‐omic predictors (genomics, proteomics, metabolomics, and demographics) of seroconversion and T1D and returned high accuracy, an AUC of 0.91 for the prediction of seroconversion based on cross‐validation of 25 case and control subjects.^10^ In this study we also undertook the evaluation of seroconversion with several core differences. The population from TEDDY is a much larger and more heterogenous. The point of seroconversion is more tightly controlled to approximately 3 and 6 months prior. Finally, the size of TEDDY allowed for independent training and validation data to evaluate the robustness of the machine learning model. One caveat to this current analysis is the predefined state of cases and controls based on matching criteria exclude potentially important factors, such as gender, clinical site, and family history, from being included in the prediction model.

In this report, we identified 42 feature candidates (SNPs, traditional risk factors, metabolites, and lipids) that, in combination, predict development of autoimmunity in increased genetic risk TEDDY participants. When interrogated, these features are associated with three biological pathways: lipid oxidation, phospholipase A2 signaling, and pentose phosphate pathway ‐ suggesting that these processes might serve as key predictive processes during development of IA. These pathways reflect processes that are regulated during an autoimmune response. These markers were identified via a data‐driven predictive model of imminent development of IA, evaluating time‐invariant risk factors in combination with time‐varying metabolic features. Models such as these may lead the field closer to the goals of precision medicine and improved understanding of the underlying biological mechanisms driving T1D.^27^ Improved understanding of the interactions between genetic factors and diet or metabolism, on the development of autoimmunity could inform new interventions to prevent or delay the onset of T1D.

## DISCLOSURE

All authors disclose no conflicts of interest.

## Supporting information


**Table S1.** Demographic and IA information on the Training and Validation sets (^a^ *t* test, ^b^χ2‐test, ^c^z‐test, ^d^Fisher0027s Exact test)
