# Supplementary material for: Prediction of the development of islet autoantibodies through integration of environmental, genetic, and metabolic markers
Source: J Diabetes. 2020 Aug 16;13(2):143–53. doi: 10.1111/1753-0407.13093 (PMC7818425; doi:10.1111/1753-0407.13093)
Supplement: Supplementary file 1 — Table S1. Demographic and IA information on the Training and Validation sets (a t test, bχ2‐test, cz‐test, dFisher0027s Exact test) [file JDB-13-143-s001.docx]

**Supplemental Table 1: Demographic and IA information on the Training and Validation sets (^a^t-test, ^b^χ2-test, ^c^z-test, ^d^Fisher’s Exact test)**

|  |  | **Training** | | **Validation** | | **p-value (Training vs. Validation)** |
| --- | --- | --- | --- | --- | --- | --- |
| **Observation** | **Value** | **Case**  **n=118** | **Control**  **n=118** | **Case**  **n=39** | **Control**  **n=39** |  |
| Age | Day | 705  ±338 | 708  ±337 | 715  ±368 | 712  ±367 | 0.87^a^ |
| Sex | Female/  Male | 59/59 | | 16/23 | | 0.33^b^ |
| FDR pairs | Count | 30 (25.4%) | | 5 (12.8%) | | 0.10^c^ |
| IA Outcome | GAD | 68 | n/a | 19 | n/a | 0.53^d^ |
|  | IA-2A | 6 | n/a | 2 | n/a |  |
|  | MIAA | 78 | n/a | 31 | n/a |  |
| Center pairs | Finland | 42 | | 16 | | 0.77^d^ |
|  | Sweden | 37 | | 15 | |  |
|  | Colorado | 15 | | 4 | |  |
|  | Washington | 11 | | 1 | |  |
|  | Germany | 7 | | 2 | |  |
|  | Georgia | 6 | | 1 | |  |
